# Hyperoside Ameliorates Renal Tubular Oxidative Damage and Calcium Oxalate Deposition in Rats through AMPK/Nrf2 Signaling Axis

**DOI:** 10.1155/2023/5445548

**Published:** 2023-03-11

**Authors:** Hongyang Tian, Qi Liang, Zhen Shi, Hang Zhao

**Affiliations:** ^1^Department of Urology, The First Affiliated Hospital of Jinzhou Medical University, Jinzhou 121000, China; ^2^Department of Urology Surgery, Dalinghe Hospital Affiliated to Medical College of Jinzhou Medical University, Jinzhou 121000, China; ^3^Department of Hepatobiliary Diseases, The First Affiliated Hospital of Jinzhou Medical University, Jinzhou 121000, China

## Abstract

**Background:**

Nephrolithiasis is a common disease that seriously affects the health and life quality of patients. Despite the reported effect of hyperoside (Hyp) against nephrolithiasis, the specific mechanism has not been clarified. Therefore, this study is aimed at investigating the effect and potential mechanism of Hyp on renal injury and calcium oxalate (CaOx) crystal deposition.

**Methods:**

Rat and cell models of renal calculi were constructed by ethylene glycol (EG) and CaOx induction, respectively. The renal histopathological damage, CaOx crystal deposition, and renal function damage of rats were assessed by HE staining, Pizzolato staining, and biochemical detection of blood and urine parameters. MTT and crystal-cell adhesion assays were utilized to determine the activity of HK-2 cells and crystal adhesion ability, biochemical detection and enzyme-linked immunosorbent assay (ELISA) to measure the levels of oxidative stress-related substances and inflammatory factors, and western blot to test the expression levels of proteins related to the AMPK/Nrf2 signaling pathway.

**Results:**

Briefly speaking, Hyp could improve the renal histopathological injury and impaired renal function, reduce the deposition of CaOx crystals in the renal tissue of rats with renal calculi, and decrease the adhesion of crystals to CaOx-treated HK-2 cells. Besides, Hyp also significantly inhibited oxidative stress response. Furthermore, Hyp was associated with the downregulation of malondialdehyde, lactate dehydrogenase, and reactive oxygen species and upregulation of superoxide dismutase activity. Additionally, Hyp treatment also suppressed inflammatory response and had a correlation with declined levels of interleukin (IL)-1*β*, IL-6, IL-8, and tumor necrosis factor. Further exploration of mechanism manifested that Hyp might play a protective role through promoting AMPK phosphorylation and nuclear translation of Nrf2 to activate the AMPK/Nrf2 signaling pathway.

**Conclusion:**

Hyp can improve renal pathological and functional damage, decrease CaOx crystal deposition, and inhibit oxidative stress and inflammatory response. Such effects may be achieved by activating the AMPK/Nrf2 signaling pathway.

## 1. Introduction

Nephrolithiasis, also known as kidney stones or renal calculi, is the third most common urinary tract problem in urology [1, 2]. Although renal calculi are benign lesion, they bring urinary tract obstruction, renal colic, and hydronephrosis to patients, and some severe cases even develop into uremia, septic shock, and tumors [3]. In fact, renal calculi seriously affect the health and quality of life of patients. It is reported that 60%-80% of renal calculi belong to calcium calculi, such as calcium oxalate (CaOx) and calcium phosphate; CaOx calculi account for 80% of all cases [4]. The 1-year recurrence rate of CaOx calculi without drug treatment is 10%, the 5-year recurrence rate is 35%, and the 10-year recurrence rate is 50% [5]. What is worse, patients with recurrent calculi are more prone to recurrence again [4]. Nephrolithiasis is influenced by various factors, such as lifestyle, diet, ethnicity, and geographic location [6], but its specific pathogenesis remains undefined. Additionally, recent studies have reported that inflammation and oxidative stress response are closely related to nephrolithiasis, and these two factors interact with each other to create a vicious cycle [7–10]. Currently, even though surgery is effective in solving most urinary calculi, it cannot prevent the formation of new calculi. Domestic and foreign scholars are committed to revealing the mechanism of renal calculi characterized with high recurrence and complex etiology, aiming to find new, effective, inexpensive, and nontoxic drugs that can not only be administered safely for a long duration but also can prevent and treat the recurrence of renal calculi.

Hyperoside (Hyp), also known as quercetin 3-O-*β*-d-galactoside, is a flavonol glycoside extracted from plants of the genera *Crataegus* or *Hypericum* [11]. Hyp has a wide range of pharmacological activities, such as anti-inflammatory, antioxidant, antidepressant, antihyperglycemic, antibacterial, antiviral, anticoagulant, and anticancer effects [11, 12]. Through the role in antioxidative stress and against inflammation [13], Hyp can alleviate cardiovascular injury and liver injury [14] caused by a variety of factors. Besides, Hyp can inhibit oxalic acid-induced oxidative damage and cytotoxicity in human renal tubular epithelial cells, but its mechanism remains to be expounded [15]. Zhu et al. proposed that intragastric administration of Hyp and quercetin could relieve renal crystal deposition and reduce urinary citrate excretion [16]. In this study, ethylene glycol (EG) and CaOx were adopted to construct a rat model of renal calculi and to induce HK-2 cells, respectively. After that, the effects of Hyp on kidney injury, CaOx crystal deposition, oxidative stress, inflammatory factors, and cell viability in the rat and cell models were explored. Additionally, the possible mechanisms of the antioxidant, anti-CaOx deposition, and anti-inflammatory functions of Hyp were further investigated. Based on the above experiments, this study provided a theoretical basis for Hyp treating nephrolithiasis.

## 2. Materials and Methods

### 2.1. Construction of Rat Model and Grouping

This study was approved by the Ethics Committee of the First Affiliated Hospital of Jinzhou Medical University (No. 2022027) and performed in accordance with the approved guidelines. EG (analytical reagent) and Hyp standards were purchased from Kunshan Horizon Chemical Co., Ltd. and Shanghai Yuanye Biotechnology Co., Ltd., respectively. A total of 40 male SD rats (age: 12 weeks; weight: about 250 g) were purchased from Shanghai Model Organisms Center and randomly divided into 4 groups (control group, Hyp group, EG group, and EG+Hyp group), with 10 rats in each group. Referring to the research of Saleem et al. [17], rat models of nephrolithiasis were established in this study. After modelling, the rats were given corresponding drugs by gavage. The EG group and the EG+Hyp group rats received drinking water with 0.75% EG, while the control group and the Hyp group rats were given an equal volume of distilled water. In the meanwhile, the Hyp group and the EG+Hyp group rats were also intragastrically administered with 50 mg/kg/d of Hyp once daily [18]. After 5 weeks, kidney tissue, blood, and urine were collected from rats in each group.

### 2.2. Histopathological Observation

After the rats were sacrificed, the left kidney tissues of three rats in each group were fixed in formalin solution, followed by paraffin embedding and sectioning (4 *μ*m thick). Hematoxylin and eosin (HE) staining was carried out according to the instructions of HE staining kit (Beijing Solarbio Science & Technology Co., Ltd., China). Upon staining, the sections were mounted with neutral resin. Subsequently, the pathological changes of kidney tissues were observed and photographed under a light microscope (CKX31, Olympus, Japan) at a magnification of ×100 and ×200.

### 2.3. Deposition of CaOx Crystals

Pizzolato staining was performed on some paraffin-embedded sections [19]. Firstly, the sections were dried in an oven at 37°C then placed in xylene for 10 min for deparaffinization. Next, they were washed with gradient ethanol then treated with 5% silver nitrate solution and 30% hydrogen peroxide for 15-30 min. Subsequently, after removal of the above working solution and rinsing samples using distilled water, 0.1% nuclear fast red staining solution was added dropwise to the sections for 1-2 min counterstaining. After that, the sections were rinsed in distilled water for 10 s, followed by dehydration and clearing. Finally, the treated sections were mounted with neutral resin. The outcomes of staining were observed under an inverted microscope, and the images were captured. The amount of CaOx crystals was analyzed using ImageJ software (version 1.8.0; National Institutes of Health, USA).

### 2.4. Detection of Biochemical Indicators

Rat kidney tissue, serum, and urine were collected. The right kidney tissues of rats were supplemented with appropriate amount of normal saline and then grind into tissue homogenates with a tissue grinder. Then, the homogenates were centrifuged (4°C, 3000 r/min for 10 min), and the supernatant was collected. Also, the blood was centrifuged (3500 rpm for 15 min) to collect serum. In the light of the instructions of biochemical assay kits (Nanjing Jiancheng Bioengineering Institute, China), serum, urine, and supernatant of the tissue homogenates were diluted into gradient solutions with different concentration, respectively. Subsequently, a biochemical automatic analyzer was utilized to determine the levels of blood urea nitrogen (BUN), creatinine (Cr), and 24 h urinary protein (24-up) in serum, oxalate (Abcam, USA) in urine, and malondialdehyde (MDA), superoxide dismutase (SOD), lactate dehydrogenase (LDH), and reactive oxygen species (ROS) in tissue supernatant [20].

### 2.5. Enzyme-Linked Immunosorbent Assay (ELISA)

The levels of TNF-*α*, interleukin (IL)-1*β*, IL-6, and IL-8 in the supernatant of tissue homogenates obtained in Section 2.4 were determined according to the instructions of the ELISA kit (Wuhan Cusabio Co., Ltd., China).

### 2.6. Cell Culture and Grouping

Human tubular epithelial cells HK-2 were purchased from the National Collection of Authenticated Cell Cultures. The samples were cultured in a DMEM/F12 (Thermo Fisher Scientific, USA) containing 10% fetal bovine serum (FBS, Gibco, USA) and 1% penicillin-streptomycin. By the way, the medium was placed in a 37°C incubator with 5% CO_2_ and changed every two days.

CaOx (analytical reagent) was purchased from Kunshan Horizon Chemical Co., Ltd. Briefly, CaOx was prepared into a solution of 0.75 mmol/L with PBS and treated with ultrasound for 15 minutes to obtain uniform crystal conditions [21]. HK-2 cells were grouped and treated as follows: the control group samples did not receive any treatment, the Hyp group samples were treated with 100 *μ*M Hyp for 4 h [22], the OX group samples were cultured with 0.75 mM CaOx medium for 48 h, and the OX+Hyp group samples were treated with 0.75 mmol/L CaOx medium for 48 h+100 *μ*M Hyp for 4 h.

### 2.7. Cell Viability Assay

The treated cells in each group were collected after trypsin digestion. The collected cells were seeded in a 96-well plate at 1 × 10^4^ cells/well and cultured in an incubator for 24 h before cell viability assay. Subsequently, 20 *μ*L MTT solution (5 mg/mL, Beyotime, China) was added to each well, and after 4 h culture, the supernatant was removed. Next, 150 *μ*L DMSO was added and mixed well with each well cells at ambient temperature for 5 min. Finally, the absorbance at 490 nm was measured by a microplate reader, with 6 duplicate wells set for each group [23].

### 2.8. Crystal-Cell Adhesion Assay

Cells and crystals were incubated together for 6 h at 4°C. Then, the supernatant aspirated, and the slides were taken out and washed by PBS three times to wash off the nonadhered crystals on the cell surface. The slides with adhered crystals and cells were digested in the mixture solution of 10 mL concentrated HNO_3_ and 1.0 mL HClO_4_ until clear. The solution was evaporated by heating, followed by cooling and addition of 3 mL ultrapure water. The concentration of Ca^2+^ ions was determined by an inductively coupled plasma emission spectrometer and then converts it into the adhered quantity of CaOx crystal. In the control group, a mixed solution of 10 mL concentrated HNO_3_ and 1.0 mL HClO_4_ was utilized to digest cells without adherent crystals (i.e., the Ca^2+^ contained in the cells themselves was subtracted) [24].

### 2.9. Detection of Protein Levels

Total proteins were extracted from rat kidney tissue and HK-2 cells, respectively, using RIPA lysate (Beijing Solarbio Science & Technology Co., Ltd., China). After quantification using BCA kit, 30 g of proteins were separated through SDS-PAGE. After that, the proteins were transferred to polyvinylidene fluoride (PVDF) membranes, followed by 1 h of blocking using 5% skimmed milk powder. Subsequently, incubation of the membranes and primary antibodies (AMPK (ab32047), p-AMPK (ab92701), HO-1 (ab305290), Nrf2 (ab76026), H3 (ab1791), and GAPDH (ab8245); Abcam; USA) was carried out at 4°C overnight. On the next day, the membranes were rinsed with TBST buffer for 10 min × 3 times, incubated with diluted secondary antibodies (Abcam; USA) for 1 h at ambient temperature, and then rinsed again. Finally, enhanced chemiluminescence agent (Beyotime, China) was used to develop proteins, with FliorChem HD2 imaging system for scanning and analyzing.

### 2.10. Statistical Analysis

All results were expressed as mean ± standard deviation (SD). SPSS 21.0 statistical software was employed for statistical analysis, *T*-test for comparison between two groups, and one-way analysis of variance for comparison among multiple groups. *P* < 0.05 acted as the criterion for significant difference.

## 3. Results

### 3.1. Hyperoside Improves Renal Pathological Injury and CaOx Crystal Deposition in Rats with Renal Calculi

**Figure 1:**
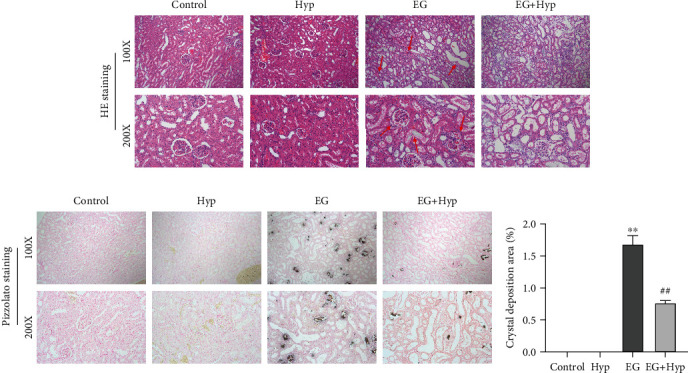
Effect of hyperoside on ethylene glycol-induced histopathological damage and calcium oxalate crystal deposition in rat kidney. (a) HE staining was used to observe the pathological damage of renal tissues; (b) Pizzolato staining was employed to assess the deposition of calcium oxalate crystals in renal tissues of rats, with black crystals representing calcium oxalate crystal deposition, ^∗∗^*P* < 0.01 vs. control group and ^##^*P* < 0.01 vs. EG group.

Firstly, we confirmed the effectiveness of a rat model of renal calculi and the effects of Hyp on renal injury and CaOx crystal deposition. The specific outcomes of each group were revealed by HE staining and Pizzolato staining. In the control group, the morphology and structure of renal tissue were normal, the basement membrane of glomerular capillaries and tubular epithelium was intact, the tubular structure was clear, and no CaOx crystal deposition was observed. In the EG group, the renal tissue presented with evident pathological damage such as glomerular edema and dilatation, inflammatory cell infiltration, and epithelial cell necrosis and shedding. Additionally, more CaOx crystal deposition was observed at the junction of renal cortex and medulla in the renal tissue of EG-treated rats. In contrast, intragastrical administration of Hyp significantly alleviated the pathological damage of rats with renal calculi and reduced CaOx crystal deposition in the renal tissue (*P* < 0.01) (Figures 1(a) and 1(b)). All above results not only suggested the successful establishment of a rat model of renal calculi by EG induction but also indicated the efficacy of Hyp in improving renal pathological injury and CaOx crystal deposition.

### 3.2. Hyperoside Alleviates Renal Function Damage in Rats with Renal Calculi

**Figure 2:**
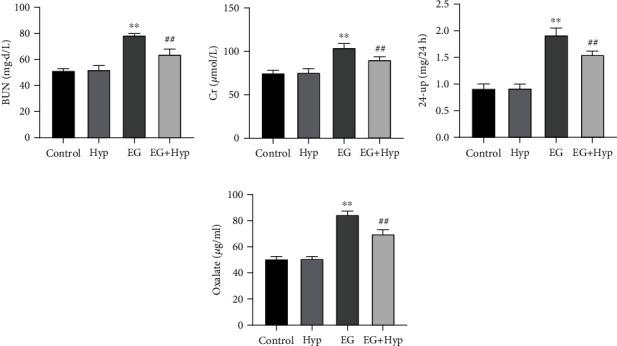
Effect of hyperoside on indicators relevant to ethylene glycol-induced renal function injury in rats. (a–d) Biochemical detection of the levels of BUN, Cr, and 24-up in the serum and oxalate in the urine of rats, ^∗∗^*P* < 0.01 vs. control group and ^##^*P* < 0.01 vs. EG group. BUN: urea nitrogen; Cr: creatinine; 24-up: 24 h urinary protein.

BUN, Cr, and 24-up in the blood and oxalate in urine are common indicators for assessing the degree of renal function damage [25]. Based on corresponding biochemical tests, the levels of these indicators were significantly increased in the EG group compared with those in the control group (*P* < 0.01), but after treatment of Hyp, an obvious decrease could be observed in these indicators (*P* < 0.01). Besides, there was no statistical difference in the above test indicators in the control group and the Hyp group (*P* > 0.05) (Figures 2(a)–2(d)). Collectively, Hyp treatment was able to significantly alleviate renal function impairment in rats with renal calculi.

### 3.3. Hyperoside Inhibits Oxidative Stress Injury and Inflammatory Response in the Kidney of Rats with Renal Calculi

**Figure 3:**
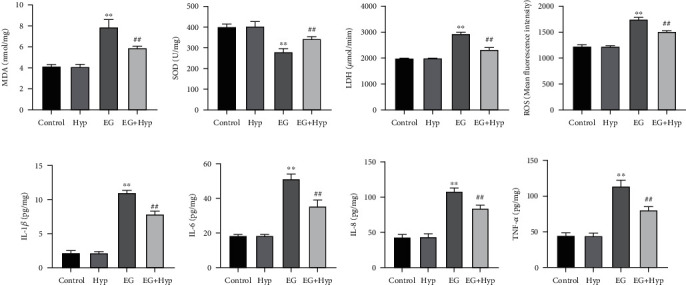
Role of hyperoside in ethylene glycol-induced oxidative stress injury and inflammatory response in rat kidney. (a–d) Biochemical detection of the levels of oxidative stress-related substances MDA (a), SOD (b), LDH (c), and ROS (d) in the kidney tissues of rats; (e–h) ELISA for detecting the levels of inflammatory cytokines IL-1*β* (e), IL-6 (f), IL-8 (g), and TNF-*α* (h) in the kidney tissues of rats. ^∗∗^*P* < 0.01 vs. control group, ^##^*P* < 0.01 vs. EG group. MDA: malondialdehyde; SOD: superoxide dismutase; LDH: lactate dehydrogenase; ROS: reactive oxygen species.

For clarifying the effects of Hyp on oxidative stress injury and inflammatory response in rats with renal calculi, we measured the levels of oxidative stress-related substances (MDA, LDH, ROS, and SOD) and inflammatory factors (IL-1*β*, IL-6, IL-8, and TNF-*α*) in the kidney tissue of rats. To be specific, there was a distinct rise in the levels of MDA, LDH, ROS, IL-1*β*, IL-6, IL-8, and TNF-*α* and an obvious decrease in the activity of SOD in the kidney tissue of rats in the EG group compared with those in the control group (*P* < 0.01). Except for significantly increased SOD activity (*P* < 0.01), the expression levels of other indicators were remarkably declined after Hyp treatment (*P* < 0.01) (Figures 3(a)–3(h)). The above indicated that Hyp treatment could make a significant reduction in oxidative stress injury and inflammatory response in the kidneys of rats with EG-induced nephrolithiasis.

### 3.4. Hyperoside Increases the Viability of HK-2 Cells Treated with Calcium Oxalate and Inhibits Crystal Adhesion

**Figure 4:**
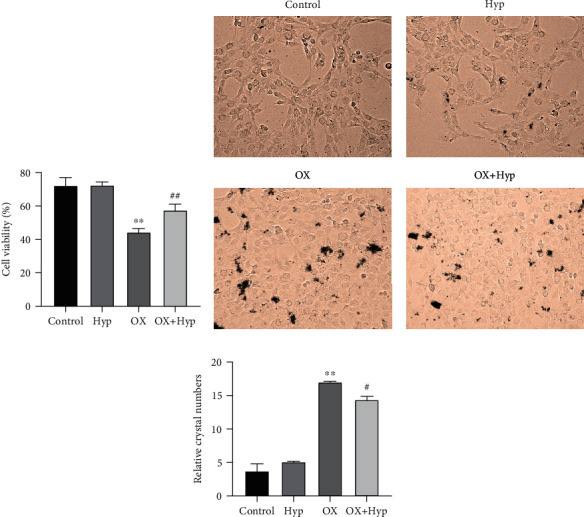
Effect of hyperoside on viability and crystal adhesion ability of calcium oxalate-treated HK-2 cells. (a) The viability of HK-2 cells was detected by MTT; (b, c) crystal-cell adhesion assay was applied to detect the adhesion of calcium oxalate crystal to HK-2 cells (200x, (b)) and statistical analysis of the number of crystals adhered to cells (c). ^∗∗^*P* < 0.01 vs. control group and ^#^*P* < 0.05 and ^##^*P* < 0.01 vs. OX group.

Subsequently, the effects of Hyp on viability and crystal adhesion ability of CaOx-treated HK-2 cells were further explored. Results of *in vitro* experiments (Figures 4(a)–4(c)) showed that compared with the control group, the viability of HK-2 cells in the OX group decreased markedly (*P* < 0.01), and the number of crystals adhered to the cells increased significantly (*P* < 0.01). In comparison with the OX group, the viability of HK-2 cells in the OX+Hyp group signaled an increment (*P* < 0.01), while the number of crystals adhered to the cells was observably decreased (*P* < 0.05). No significant difference was observed in the corresponding phenotype between the control group and the Hyp group (*P* > 0.05). Taken together, Hyp could increase the viability and inhibit the crystal adhesion ability of CaOx-treated HK-2 cells.

### 3.5. Hyperoside Alleviates Oxidative Stress and Inflammatory Response in HK-2 Cells Treated with Calcium Oxalate

**Figure 5:**
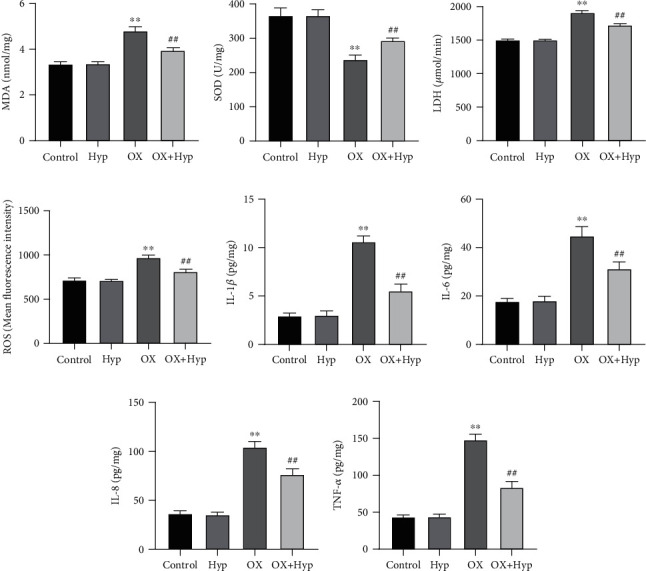
Effect of hyperoside on oxidative stress and inflammatory response in calcium oxalate-treated HK-2 cells. (a–d) Biochemical tests for MDA (a), SOD (b), LDH (c), and ROS (d) levels in cells; (e–h) ELISA detection of IL-1*β* (e), IL-6 (f), IL-8 (g), and TNF-*α* (h) levels in cells, ^∗∗^*P* < 0.01 vs. control group and ^##^*P* < 0.01 vs. OX group. MDA: malondialdehyde; SOD: superoxide dismutase; LDH: lactate dehydrogenase; ROS: reactive oxygen species.

*In vivo* experiments proved that Hyp could inhibit ROS and inflammatory response in the kidney of rats with renal calculi. In this study, we further clarified if Hyp had the same antioxidant and anti-inflammatory effects on CaOx-treated HK-2 cells. The results indicated that the levels of MDA, LDH, ROS, IL-1*β*, IL-6, IL-8, and TNF-*α* in the cells of the OX group went up markedly, whereas the activity of SOD went down obviously, relative to the control group (*P* < 0.01). The intervention of Hyp could observably lower the levels of MDA, LDH, ROS, IL-1*β*, IL-6, IL-8, and TNF-*α* in HK-2 cells treated with CaOx (*P* < 0.01), and increase the activity of SOD (*P* < 0.01). The difference between the control group and the Hyp group was not statistically significant (*P* > 0.05) (Figures 5(a)–5(h)). In a nutshell, Hyp could alleviate oxidative stress and inflammatory response in HK-2 cells treated with CaOx.

### 3.6. Hyperoside Activates the AMPK/Nrf2 Signaling Pathway in the Kidney Tissue of Renal Calculi Rats and the Calcium Oxalate-Treated HK-2 Cells

**Figure 6:**
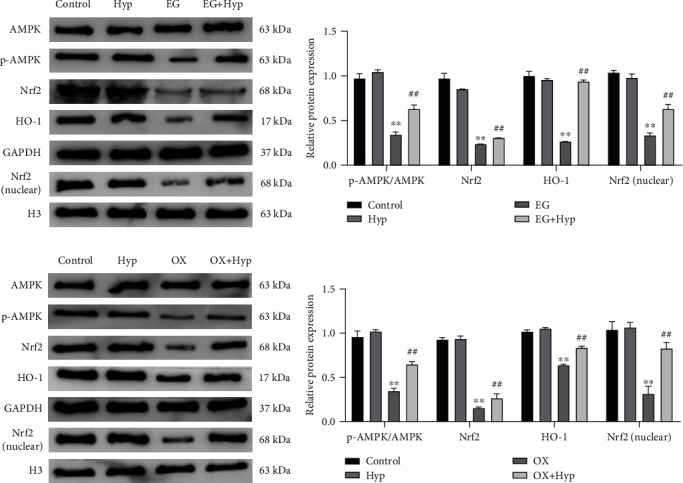
Effect of hyperoside on AMPK/Nrf2 signaling pathway in kidney tissue of nephrolithiasis rats and in HK-2 cells treated with calcium oxalate. (a–d) Western blot and quantitative analyses for the protein expression levels of AMPK, p-AMPK, Nrf2, HO-1, and nuclear Nrf2 in the kidney tissues of rats (a, b) and in HK-2 cells treated with CaOx (c, d). ^∗∗^*P* < 0.01 vs. control group and ^##^*P* < 0.01 vs. EG group or OX group.

Studies have revealed that the AMPK/Nrf2 signaling pathway is closely linked to renal inflammation and oxidative stress in diabetic rats [26]. However, whether Hyp plays a protective role in renal injury caused by renal calculi by regulating this pathway remained to be explored. To clarify this speculation, we examined the expression levels of the AMPK/Nrf2 signaling pathway-related proteins (p-AMPK, AMPK, Nrf2, and HO-1) in kidney tissues of nephrolithiasis rats and HK-2 cells treated with CaOx (Figures 6(a)–6(d)). Both *in vitro* and *in vivo* experiments demonstrated that, in comparison with the control group, p-AMPK was evidently inhibited in the kidney tissue of the EG group and the cells of the OX group; besides, the protein expression levels of total Nrf2, nuclear Nrf2, and HO-1 were all remarkably declined (*P* < 0.01) in these two groups. Hyp treatment increased the phosphorylation level of AMPK in the kidney of rats with renal calculi and HK-2 cells treated with CaOx; also, it augmented the nuclear translocation of Nrf2 and HO-1 expression (*P* < 0.01). The difference between the control and Hyp groups was not statistically significant (*P* > 0.05). The above experiment results suggested that Hyp could reverse AMPK/Nrf2 activity inhibition in the CaOx-treated HK-2 cells and kidney tissue from nephrolithiasis rats.

## 4. Discussion

Nephrolithiasis is a common urological disease. Drugs such as sodium oxalate, ammonium oxalate, hydroxy-l-proline, EG, and glycolic acid are usually employed to induce acute or chronic hyperoxaluria to establish the model of rats with CaOx nephrolithiasis [27]. The occurrence of renal stone depends not so much on the formation of crystals but on their retention in the kidney. It is reported that crystal retention is predominantly caused by the adherence of crystals to the epithelial cells lining the renal tubules [28]. The severity of nephrolithiasis can be assessed by the adhesion of CaOx crystals to the epithelial cells. In this study, drinking water with 0.75% EG was applied to establish a rat model of CaOx nephrolithiasis. HE and Pizzolato staining results of pathological tissue sections (glomerular edema and dilatation, inflammatory cell infiltration, and CaOx crystal deposition) and biochemical tests of blood and urine indicators (BUN, Cr, 24-up, and oxalate) proved that we successfully constructed a rat model of CaOx nephrolithiasis.

Rats with EG-induced renal calculi present with a systemic increase in ROS; increased ROS will gather in the kidney through blood circulation and disrupt the balance of redox in the body, thereby causing renal injury [29]. SOD, whose level can reflect the body's capability against oxidative stress, plays a role in scavenging excessive ROS [30]. MDA, as a product of fatty acid peroxidation, can reflect the degree of cellular peroxidation in the body [31]. LDH is able to convert glyoxylate to oxalic acid in the body and induce recurrent urinary calculi and loss of renal function, thereby resulting in renal failure [32]. In this study, Hyp treatment could effectively relieve the renal function damage and histopathological damage caused by renal calculi and CaOx crystal deposition. Additionally, Hyp exerted an antioxidant function in the models of nephrolithiasis, indicated by increased SOD activity and decreased expression of MDA, LDA, and ROS after Hyp treatment. Similarly, Chen et al. also discovered that Hyp was capable of notably reducing the levels of oxidative stress-related substances such as ROS and LDH in oxalic acid-treated HK-2 cells [15].

A study has manifested that, due to the accumulation of a large number of macrophages in a rat model of renal calculi, inflammatory factors released by macrophages may be related to the formation of CaOx crystals [33]. Liu et al. reported that with the increase of the Ox/Ca ratio, the amount of CaOx crystals induced by cells increased, the degree of crystal aggregation increased, and the toxicity to the cells increased [34]. HK-2 cells in a supersaturated CaOx solution can induce CaOx crystal formation and then cause damage to the cells. We explored the effects of Hyp on viability and crystal adhesion ability of CaOx-treated HK-2 cells and found that Hyp could increase the viability and inhibit the crystal adhesion ability of CaOx-treated HK-2 cells. In this study, Hyp intervention exhibited a notable decrease in the levels of IL-1*β*, IL-6, IL-8, and TNF-*α* in cell and rat models, suggesting the anti-inflammatory role of Hyp in nephrolithiasis. Our findings are consistent with the existing reports regarding the anti-inflammatory effect of Hyp. For example, Huang et al. revealed that Hyp inhibited lipopolysaccharide- (LPS-) induced inflammatory response in HT22 cells by suppressing the levels of IL-1*β*, IL-6, IL-8, and TNF-*α* [35]; in the meanwhile, Zhou et al. also discovered that Hyp inhibited IL-1*β*, IL-6, and TNF*α* to play an anti-inflammatory role in LPS-induced endothelial cells [36].

AMPK is a serine/threonine protein kinase that is mainly involved in the regulation of glucose, lipid, and energy metabolism *in vivo*. Studies have reported that the AMPK activation can inhibit the inflammatory response and oxidative stress response of mouse macrophages [37]. Nrf2 is a key molecule that regulates the transcription of antioxidant factors in the body, which has been proved to be activated by AMPK [38]. AMPK/Nrf2 plays a critical role in kidney injury. For instance, the prescription of dispersing blood stasis and dredging collateral (HTR) can protect the kidney by inhibiting renal oxidative stress and inflammation by activating the AMPK/Nrf2 signaling pathway [25]. Hyp can also improve the endogenous antioxidant and detoxification functions of kidney cells through the Nrf2/HO-1/quinone oxidoreductase 1 (NQO1) pathway [15]. Moreover, Gao et al. revealed that Hyp was able to increase the phosphorylation level of AMPK in particulate matter-induced lung injury [39]. Therefore, we speculated that Hyp may exert antioxidant and anti-inflammatory functions in nephrolithiasis through AMPK/Nrf2. Further experiments indeed demonstrated that the AMPK/Nrf2 pathway was remarkably inhibited in the rat and cell models of nephrolithiasis. Specifically, after Hyp intervention, the AMPK phosphorylation, nuclear translocation level of Nrf, and HO-1 expression were evidently increased. These findings suggest that Hyp can promote AMPK phosphorylation and nuclear translocation of Nrf2 to activate the AMPK/Nrf2 signaling pathway. Unfortunately, the antioxidant and anti-inflammatory effects of Hyp on the kidney injury were not explored by pathway reagents in this study, so further experimental validation is needed.

There are some limitations in the study. First, long-term application of high-dose Hyp should be avoided in clinical practice due to its renal toxicity [40]. However, the concentration gradient of Hyp was not set when exploring the effect of Hyp in the models of nephrolithiasis, so the optimal dose and whether its effect was concentration-dependent were unclear. Notably, an *in vitro* study by Chen et al. presented that the protective effect of Hyp on oxalic acid-induced OS injury in HK-2 cells was concentration-dependent [15]. Second, concerning the possible molecular mechanism of Hyp, we only examined the AMPK/Nrf2 pathway activity, and more clear mechanisms need further experimental exploration.

## 5. Conclusion

To summarize, Hyp can not only improve renal pathological injury, functional injury, and CaOx crystal deposition in rats with renal calculi but also can inhibit their oxidative stress and inflammatory response, according to the results of *in vivo* experiments. Additionally, Hyp can inhibit the cell activity, crystal adhesion ability, and oxidative stress and inflammatory response in CaOx-related HK-2 cells. Hyp may promote the AMPK phosphorylation and nuclear translation of Nrf2 to activate the AMPK/Nrf2 signaling pathway, thereby exerting protective functions in renal injury.

## Data Availability

The datasets generated during and/or analyzed during the current study are available from the corresponding author on reasonable request.

## References

[1] Merchant M. L., Cummins T. D., Wilkey D. W. (2008). Proteomic analysis of renal calculi indicates an important role for inflammatory processes in calcium stone formation. *American Journal of Physiology Renal Physiology*.

[2] Chauhan N., Kumar D., Kasana M. S. (2009). Medicinal plants of Muzaffarnagar district used in treatment of urinary tract and kidney stones. *Indian Journal of Traditional Knowledge*.

[3] Xu H., Chen D. Y., Liu Y. D. (2019). Effect of citric acid on Tamm-Horsfall protein and MCP-1 in urine of rats with kidney stone induced by CNPs. *Heilongjiang Medicine and Pharmacy*.

[4] Evan A. P. (2010). Physiopathology and etiology of stone formation in the kidney and the urinary tract. *Pediatric Nephrology*.

[5] Li Y., Zhang J., Liu H. (2019). Curcumin ameliorates glyoxylate-induced calcium oxalate deposition and renal injuries in mice. *Phytomedicine*.

[6] Sorensen M. D., Hsi R. S., Chi T. (2014). Dietary intake of fiber, fruit and vegetables decreases the risk of incident kidney stones in women: a Women's Health Initiative report. *The Journal of Urology*.

[7] Khan S. R., Pearle M. S., Robertson W. G. (2016). Kidney stones. *Nature Reviews Disease Primers*.

[8] Mulay S. R., Kulkarni O. P., Rupanagudi K. V. (2013). Calcium oxalate crystals induce renal inflammation by NLRP3-mediated IL-1*β* secretion. *The Journal of Clinical Investigation*.

[9] Khan S. R. (2013). Reactive oxygen species as the molecular modulators of calcium oxalate kidney stone formation: evidence from clinical and experimental investigations. *The Journal of Urology*.

[10] Evan A. P., Lingeman J. E., Worcester E. M. (2010). Renal histopathology and crystal deposits in patients with small bowel resection and calcium oxalate stone disease. *Kidney International*.

[11] Wang C. Y., Li J. (2019). Myocardial protective effect and mechanism of hyperoside in severely burned rats. *Journal of Shanxi Medical University*.

[12] Bahmani M., Baharvand-Ahmadi B., Tajeddini P., Rafieian-Kopaei M., Naghdi N. (2016). Identification of medicinal plants for the treatment of kidney and urinary stones. *Injury Prevention*.

[13] Wang X., Liu Y., Xiao L. (2018). Hyperoside protects against pressure overload-induced cardiac remodeling via the AKT signaling pathway. *Cellular Physiology and Biochemistry*.

[14] Xing H.-Y., Liu Y., Chen J.-H., Sun F.-J., Shi H.-Q., Xia P.-Y. (2011). Hyperoside attenuates hydrogen peroxide-induced L02 cell damage via MAPK- dependent Keap_1_-Nrf_2_-ARE signaling pathway. *Biochemical and Biophysical Research Communications*.

[15] Chen Y., Ye L., Li W., LiF D. (2018). Hyperoside protects human kidney-2 cells against oxidative damage induced by oxalic acid. *Molecular Medicine Reports*.

[16] Zhu W., Xu Y. F., Feng Y. (2014). Prophylactic effects of quercetin and hyperoside in a calcium oxalate stone forming rat model. *Urolithiasis*.

[17] Saleem U., Ahmad N., Shah M. A., Anwar F., Ahmad B. (2020). Anti-urolithiatic activity of Salvia hispanica L. seeds in ethylene glycol induced urolithiasis rat's model. *Anais da Academia Brasileira de Ciências*.

[18] Chen X., Famurewa A. C., Tang J., Olatunde O. O., Olatunji O. J. (2022). Hyperoside attenuates neuroinflammation, cognitive impairment and oxidative stress via suppressing TNF-*α*/NF-*κ*B/caspase-3 signaling in type 2 diabetes rats. *Nutritional Neuroscience*.

[19] Yang X., Liu H., Ye T. (2020). AhR activation attenuates calcium oxalate nephrocalcinosis by diminishing M1 macrophage polarization and promoting M2 macrophage polarization. *Theranostics*.

[20] Liu Y. D., Yu S. L., Wang R. (2019). Rosiglitazone Suppresses Calcium Oxalate Crystal Binding and Oxalate-Induced Oxidative Stress in Renal Epithelial Cells by Promoting PPAR- *γ* Activation and Subsequent Regulation of TGF- *β* 1 and HGF Expression. *Oxidative Medicine and Cellular Longevity*.

[21] Song Q., Liao W., Chen X. (2021). Oxalate activates autophagy to induce ferroptosis of renal tubular epithelial cells and participates in the formation of kidney stones. *Oxidative Medicine and Cellular Longevity*.

[22] Zhou J., Zhang S., Sun X., Lou Y., Yu J. (2021). Hyperoside protects HK-2 cells against high glucose-induced apoptosis and inflammation via the miR-499a-5p/NRIP1 pathway. *Pathology Oncology Research*.

[23] Lai Y., Liang X., Zhong F. (2019). Allicin attenuates calcium oxalate crystal deposition in the rat kidney by regulating gap junction function. *Journal of Cellular Physiology*.

[24] Qiong-Zhi G., Chong-Yu Z., Jian-Ming O. (2016). Effect of crystal size of calcium oxalate dihydrate on cytotoxicity of African green monkey kidney epithelial cells. *Journal of Inorganic Materials*.

[25] Moe O. W. (2006). Kidney stones: pathophysiology and medical management. *Lancet*.

[26] Li Y., Guo S., Yang F., Liu L., Chen Z. (2021). Huayu Tongluo recipe attenuates renal oxidative stress and inflammation through the activation of AMPK/Nrf2 signaling pathway in streptozotocin- (STZ-) induced diabetic rats. *Evidence-based Complementary and Alternative Medicine*.

[27] Khan S. R. (1997). Animal models of kidney stone formation: an analysis. *World Journal of Urology*.

[28] Asselman M., Verkoelen C. F. (2002). Crystal-cell interaction in the pathogenesis of kidney stone disease. *Current Opinion in Urology*.

[29] Thamilselvan S., Byer K. J., Hackett R. L., Khan S. R. (2000). Free radical scavengers, catalase and superoxide dismutase provide protection from oxalate-associated injury to LLC-PK1 and MDCK cells. *The Journal of Urology*.

[30] Sato Y., Kajiyama S., Amano A. (2008). Hydrogen-rich pure water prevents superoxide formation in brain slices of vitamin C-depleted SMP30/GNL knockout mice. *Biochemical and Biophysical Research Communications*.

[31] Roy S., Sable P., Khaire A., Randhir K., Kale A., Joshi S. (2014). Effect of maternal micronutrients (folic acid and vitamin B_12_) and omega 3 fatty acids on indices of brain oxidative stress in the offspring. *Brain and Development*.

[32] Zheng R., Fang X., Chen X. (2020). Knockdown of lactate dehydrogenase by adeno-associated virus-delivered CRISPR/Cas9 system alleviates primary hyperoxaluria type 1. *Clinical and Translational Medicine*.

[33] Kanlaya R., Sintiprungrat K., Chaiyarit S., Thongboonkerd V. (2013). Macropinocytosis is the major mechanism for endocytosis of calcium oxalate crystals into renal tubular cells. *Cell Biochemistry and Biophysics*.

[34] Liu H., Zou G. J., SunJ X. Y., Ouyang M. (2020). Differences of growth and cytotoxicity of calcium oxalate crystals formed on HK-2 cells under different oxalic acid/calcium ratios. *Chemical Journal of Chinese Universities*.

[35] Huang J., Zhou L., Chen J. (2021). Hyperoside Attenuate Inflammation in HT22 Cells via Upregulating SIRT1 to Activities Wnt/ *β* -Catenin and Sonic Hedgehog Pathways. *Neural Plasticity*.

[36] Zhou Y. Q., Zhao Y. T., Zhao X. Y. (2018). Hyperoside suppresses lipopolysaccharide-induced inflammation and apoptosis in human umbilical vein endothelial cells. *Current Medical Science*.

[37] Huang B. P., Lin C. H., Chen H. M., Lin J. T., ChengS Y. F., Kao H. (2015). AMPK activation inhibits expression of proinflammatory mediators through downregulation of PI3K/p38 MAPK and NF-*κ*B signaling in murine macrophages. *DNA and Cell Biology*.

[38] Peng M., Qiang L., Xu Y., Li C., Li T., Wang J. (2019). Inhibition of JNK and activation of the AMPK-Nrf2 axis by corosolic acid suppress osteolysis and oxidative stress. *Nitric Oxide*.

[39] Gao Y., Fan X., Gu W., Ci X., Peng L. (2021). Hyperoside relieves particulate matter-induced lung injury by inhibiting AMPK/mTOR-mediated autophagy deregulation. *Pharmacological Research*.

[40] Xu S., Chen S., Xia W., Sui H., Fu X. (2022). Hyperoside: a review of its structure, synthesis, pharmacology, pharmacokinetics and toxicity. *Molecules*.

